# Trends of hospital admissions and mortality of patients with dementia: descriptive study in Lombardy

**DOI:** 10.1093/eurpub/ckac131.139

**Published:** 2022-10-25

**Authors:** L Blandi, A Amorosi, P Bertuccio, A Odone

**Affiliations:** 1 Public Health, Università di Pavia, Pavia, Italy; 2 Welfare Directorate, Regione Lombardia, Milan, Italy

## Abstract

**Background:**

In 2017 the amount of people globally affected by dementia was estimated about 50 million and is predicted to increase to 132 million by 2050. Coping with dementia is one of the most important challenges of governmental organizations’ agenda. The objective of this study is to analyse the hospital admissions trends and monitor the overall mortality in a population of older patients with dementia over the last two decades in northern Italy.

**Methods:**

This study is based on the healthcare utilization database of the Lombardy region (Italy), considering on hospital discharge records and death registry flows. Primary or secondary diagnosis at admission of dementia was coded according to the ICD9-CM. We carried out descriptive analyses of hospital admissions’ data from 1 Jan 1999 to 31 Dec 2020 of older patients aged 65 or more. We then conducted a temporal analysis of mortality rate over the study period.

**Results:**

A total of 15,683,024 hospital admissions occurred during the study period. Over the last two decades, the prevalence of dementia among patients admitted to acute care hospitals remained around 1.1-1.3%. Considering the total of 183,268 patients with dementia over the study period, the average age at admission increased from 80.2 in 1999 to 83.3 years old in 2020, whereas annual mortality rate increased from about 1,950 to 3,230 deaths per 10,000 person-years. The mortality rate ratio of patients with versus without dementia fluctuated between 1.28 and 1.70.

**Conclusions:**

Our findings suggest that there is an ever-greater appropriateness of hospitalizations over the last two decades, supported by out-of-hospital care that led patients to hospitalization in increasingly late and serious phases of the disease. The present study has a great future potential as well as limitations, due to the dependence on a correct coding of cases by clinicians according to the ICD9-CM system.

**Key messages:**

• We observed an increased mortality among older people with dementia admitted to hospital over the last two decades.

• Our descriptive study, based on the Lombardy regional healthcare database, provides evidence of an increasing appropriateness of hospitalizations.

